# Body Composition Characteristics of Type 1 Diabetes Children and Adolescents: A Hospital-Based Case-Control Study in Uganda

**DOI:** 10.3390/children9111720

**Published:** 2022-11-09

**Authors:** Jonathan Nsamba, Priscilla Eroju, Fotios Drenos, Elezebeth Mathews

**Affiliations:** 1 Department of Public Health & Community Medicine, Central University of Kerala, Tejaswini Hills, Periye 671316, India; 2 Department of Life Sciences, College of Health, Medicine and Life Sciences, Brunel University London, Kingston Lane, London UB8 3PH, UK; 3 Makerere University–John Hopkins University (MUJHU) Collaboration, Kampala P.O. Box 23491, Uganda

**Keywords:** body composition, impedance, phase angle, children, adolescents, Uganda

## Abstract

**Background:** Changes in body composition have been suggested as an intractable effect of Type 1 Diabetes Mellitus and its management. This study aims to compare body composition characteristics in a sample of young children and adolescents with Type 1 Diabetes Mellitus with healthy controls. **Methods:** In this case–control study, body composition was assessed using bioelectrical impedance among 328 participants. Anthropometric measurements included weight, height, upper arm, hip, and waist, circumferences; biceps; triceps; and subscapular and suprailiac skinfolds. From raw Bioelectrical impedance data, we calculated the impedance, phase angle, and height normalised resistance and reactance to assess body composition. Analysis of variance accounting for paired blocks was used to compare the two matched groups, while an independent Student’s *t*-test was used for intragroup comparisons among cases. **Results:** Waist Hip Ratio, biceps, triceps, subscapular and suprailiac skinfolds were higher among cases than in controls. Cases showed a higher Fat Mass Index, higher fasting blood glucose and higher glycated haemoglobin. Cases also had a higher mean value of resistance (*p* = 0.0133), and a lower mean value of reactance (*p* = 0.0329). Phase angle was lower among cases than in controls (*p* < 0.001). **Conclusion:** Our diabetic children showed higher levels of adiposity than controls. The observed differences in body composition are explained by differences in the fat-mass index. Abdominal fat accumulation was associated with poor glycaemic control and a lower phase angle.

## 1. Introduction

There is increasing interest in assessing the body composition of children and adolescents with diabetes. Scientific evidence stresses that children with Type 1 Diabetes Mellitus (T1DM) have a greater preponderance of weight gain than their closely matched healthy peers [1,2,3,4]. The pathophysiology of weight changes remains largely uncertain, and since the quality of weight gain hugely depends on its composition, we are unsure whether it’s the fat or fat-free mass compartments. It has been documented that poor dietary intake, coupled with a lack of physical activity and exogenous insulin therapy administration, are responsible for weight gain among people with diabetes [5,6].

The assessment of body composition is a critical approach in the early identification of changes at a cellular level [7], making it a vital tool in assessing health status since alterations in body compartments of fat and fat-free mass have health risk implications [8]. Body composition measurements can inform the clinical diagnosis of disease, improve prognosis, and facilitate the early assessment of adverse metabolic outcomes [8]. Understanding the body composition of diabetic children is vital for monitoring response to treatment and managing resulting weight changes [9].

Body composition can be accurately assessed through air-displacement plethysmography, Magnetic Resonance Imaging (MRI), Dual-energy X-ray Absorptiometry (DXA) and deuterium dilution; however, the applicability of these methods in clinical and field settings is difficult due to their cost [2,7]. On the other hand, Bioelectrical Impedance (BIA) is a relatively cheaper, non-invasive, reliable and widely accepted method of body composition estimation [10,11], even among people with diabetes [10].

BIA is based on the principle that body tissues are composed of fluid electrolytes that conduct electric currents [12]. BIA involves passing a low voltage current across the body with the resistance, corresponding to opposition to the current flow and reactance, corresponding to the capacitance of body tissues registered [13]. The values of resistance and reactance are thus converted into corresponding values of body fat mass, fat-free mass, muscle mass and total body water through predictive regression equations [14]. However, one of the challenges with using BIA among young populations is the calculation of total body water [15]. These equations make predictions of body compartments considering a fixed 73% hydration level of tissues, but in metabolic disease states like diabetes characterized by abnormal hydration [16,17], these algorithms produce inaccurate estimates [15]. Secondly, predictive equations are sex and population-specific [18], thus limiting their use across different ethnic populations [19], for instance our study population. To counter these limitations [12], we assessed body composition with the use of central adiposity anthropometric measures while standardizing the raw values of reactance and resistance with height [20]. We further calculated the phase angles [21], a vital clinical tool for monitoring health, nutrition and treatment prognosis.

The evaluation of body composition among diabetic children is vital in informing clinical diagnosis, early assessment of cardiometabolic risk and optimizing treatment [22]. In this study, we compared body composition parameters among cases and controls to investigate for any differences. Diabetes and its management have been associated with alterations in body composition parameters [22] among diverse groups of children in the world. Whether these observations are similarly replicated among our group of children is not currently known. We aimed to explore if diabetic children in Uganda similarly showed weight and body fat alterations despite a high burden of malnutrition, infectious diseases and other environmental-related challenges like food insecurity and famine.

## 2. Materials and Methods

### 2.1. Study Design

A hospital-based case–control study was conducted between March 2021 and January 2022, with data collected from three study sites in Uganda (St Francis Hospital Nsambya, Mulago National Referral Hospital and Wakiso Health Centre IV) located in the central region of Uganda.

### 2.2. Study Population

A power analysis for Analysis of Variance and an independent *t*-test was done in R-software using the “pwr” package to determine the sufficient sample size with an alpha = 0.05, and power of 80%. Based on these assumptions, the sample size was 328 participants with 164 T1DM cases recruited and matched in a 1:1 ratio with 164 controls. The sample size was sufficient to detect small differences between the two groups and to prevent type II error. Cases were obtained from various clinics under the Changing Diabetes in Children (CDiC) program. The CDiC program is a public-private partnership aimed at increasing access to diabetes care for children with T1DM. Further details about the program have been previously reported [23].

Cases were children and adolescents between 6 and < 18 years with clinically diagnosed T1DM. Cases had to have been active with their hospital appointments and receiving insulin therapy for at least three months before the date of data collection. Cases were purposively recruited as they came to attend their hospital visits in a probability-to-size sampling approach based on the population of the study site. Controls comprised healthy children and adolescents matched for age, sex and geographical location. For each patient, one healthy non-diabetic control was recruited. Control subjects were either siblings if they were of the same sex and within an age difference of less than two years. If this criterion was not satisfied, a corresponding control was recruited from the neighbourhood community matched for sex and age. There were no significant differences in sex, age, weight, height, or geographical location among the two groups of our study participants.

### 2.3. Anthropometry

The body measurements of each participant were measured in the morning after an overnight fast following standard protocols [24], and all measurements (height, weight, body circumferences & skinfolds, BIA, fasting blood glucose, and HbA1c were done on the same day.

Participants were measured wearing light clothing and barefoot. Height was determined to the nearest 0.1 cm using a fixed stadiometer on the wall, with the participant standing erect and barefoot. Sitting height and leg length were recorded to the nearest 0.1 cm. Bodyweight was measured on a Seca portable electronic scale (Model 874, Hamburg, Germany) to the nearest 0.1 kg. Waist and hip circumferences were measured in the midline between the lower rib margin and the iliac crest and the widest diameter over the greater trochanters, respectively, with the subjects standing with their heels together. A waist-to-hip ratio (WHR) was then calculated. A Seca 203 ergonomic measuring tape measured the waist and hip circumferences. Mid Upper Arm Circumference was measured using a Uganda Ministry of Health-approved tape at the midpoint between the acromion process of the Scapula and the most distal point on the olecranon process. Skinfold circumferences were measured using a Harpenden Skinfold Caliper (Model SFCH80, India) at biceps, triceps, subscapular and suprailiac sites and recorded to the nearest 0.1 mm. We calculated the Technical Error of Measurement for the different anthropometric values to ensure precision and validity of the measurements as recommended by the International Society for Advancement in Kinanthropometry [25].

### 2.4. Bioelectrical Impedance

Body composition was measured using a duo frequency non-segmental Bio-electrical Impedance analyser with participants standing on bare feet in a supine position on the posterior electrode base of the body composition analyser machine (Tanita DC-430MA TANITA Corporation, Tokyo, Japan). Resistance (R, in Ohm, Ω) and reactance (XC, in Ω) raw values at 50-kHz frequency were recorded [26]. Impedance was calculated as √R2 + Xc2 and the phase angle as the arctangent of (Xc/R) [17]. To ensure accuracy, the first author undertook all measurements in the morning before children had consumed any fluids which would alter their hydration status.

### 2.5. Biochemical Assessments

Fasting blood glucose was measured using a glucometer (Accu-Check^®^ Active, Mumbai, India) after an overnight fast. Pulse rate, systolic & diastolic blood pressures were measured by AccuMed^®^ wrist blood pressure monitor. Glycaemic control was measured by HbA1c analyser BioHermes^®^ (A1C EZ2.0, USA), and HbA1c <  7.5% (<58 mmol/mol) was considered good glycaemic control as recommended by the East African Diabetes Study Group for Ugandan children and adolescents [27]. Furthermore, dietary diversity scores of food group distribution were analysed from 24 h recalls. Physical activity was measured by five questions adapted from the World Health Organisation Global Physical Activity Questionnaire [28]. Adherence was self-reported by participants indicating whether they had challenges sticking to their treatment regimen.

### 2.6. Data Analysis

Descriptive statistics were calculated as frequencies, means and standard deviations. Pearson correlation (r) was used to assess correlations between body composition parameters. Height normalized indices of Fat Mass, and Fat-Free Mass were calculated by dividing individual masses by the height in square metres (kg/m^2^). Three age groups were derived: prepubertal (6–10 years), pubertal (10.1–14.9 years) and post-pubertal (15–17.9 years). Despite overlaps reported across populations, especially in terms of gender differences, these age categories were largely agreed upon to account for the differing effect of puberty on body composition. A two-way analysis of variance was used to compare the mean of outcomes between cases and controls while controlling separately for interactions of age categories, glycaemic control and level of adherence by examining interaction plots.

Body composition characteristics of patients were compared to those of controls using an Analysis of Variance, taking into account the matching pairs using a block design while controlling for age, sex, geographical location, and in many cases, relatedness between cases and controls. For intragroup comparisons, we used the independent samples t-test with unequal variances to test for differences within the groups. Interaction of age, type of insulin, adherence levels and levels of glycaemic control was assessed using a two-way factor Analysis of variance. Before conducting the analysis, all normality assumptions were checked using Q-Q plots, and variables (Fat Mass Index, fasting blood glucose, Glycated haemoglobin, Biceps, Triceps, Subscapular, Suprailiac skinfolds) whose distribution was skewed were log-transformed. The significance level adopted was 5% (*p* ≤ 0.05) without multiple testing corrections. Analyses were performed using R software, version 4.0.2 (R Foundation for Statistical Computing, Vienna, Austria).

## 3. Results

The study population comprised 328 participants. Healthy participants in the control group were matched with controls; therefore, there were no statistically significant differences in age (*p*-value = 0.34), sex (*p*-value = 0.83), geographical location (*p*-value = 0.27) between the diabetic and non-diabetic participants. Demographic profiles of both cases and controls are summarised in Table 1.

### 3.1. Medical History of Cases

Cases were recruited from the diabetic clinics of three regional hospitals in Uganda, with 73 (44.5%) from Mulago National Referral Hospital, 83 (50.6%) from St Francis Hospital Nsambya and 08 (4.9%) from Wakiso Health Centre IV. Most of the patients, 126 (76.8%), were up to date with their appointments with no missed hospital visits within the previous three months. All diabetic participants were active on insulin hormonal therapy (n = 164 (100%)), with 109 (66.5%) using long-acting insulin (Insulatard) and fast-acting insulins (Actrapid) while 55 (33.5%) on Mixtard. Majority of the cases, 137 (83.54%) had an acceptable level of adherence to insulin therapy. Of all the diabetic participants, 79 (48.2%) had been using insulin for <2 years, 61 (37.2%) for 2–4.99 years, and 24 (14.6%) for five years and above. Further details are summarised in Table 2.

Compared to controls, cases showed statistically significant differences in anthropometric indicators (Table 3). The results indicated that diabetic participants had higher means of Waist Hip Ratio, biceps, triceps, subscapular and suprailiac skin folds than their healthy counterparts. Sex disaggregated means show higher values of anthropometric measures among females than their male counterparts (Table 3).

Patients with Type 1 Diabetes Mellitus showed bioelectrical and metabolic characteristics statistically different from those of controls (Table 4). In particular, they showed a higher Fat Mass Index, fasting blood glucose and glycated haemoglobin. Cases had higher values of systolic and diastolic blood pressure. Study participants showed no statistical differences in group means regarding their dietary intake and Fat-Free Mass Index across groups.

### 3.2. Bioelectrical Characteristics

Table 5 summarises the resistance (R), reactance (Xc), Impedance, Phase angle and height normalised R and Xc between the two groups. Particularly, diabetic participants had a higher mean value of resistance (*p* = 0.0133), and a lower mean value of reactance (*p* = 0.0329). The phase angle, a vital indicator for nutrition status and general health status, was lower among cases than in controls (*p* < 0.001).

### 3.3. Intragroup Comparisons

Results of an independent Student’s *t*-test among diabetic participants showed Fat Mass Index to differ significantly between males and females (mean difference = 0.52, *p* < 0.001). Females showed higher values of Fat Mass Index than their male counterparts, while males had higher Fat-Free Mass Index (*p* = 0.0259). Among female cases, participants who reported having started menstruation had higher values of Fat Mass Index than those who had not (mean difference = 0.86, *p* < 0.001).

We further investigated how body composition differs among cases with good glycaemic control (HbA1c < 7.5%) and those with poor control (HbA1c ≥ 7.5). Results (Table 6) showed that diabetic participants with good glycaemic control (n = 89) had higher values of Fat-Free Mass (*p* = 0.0143) than those with poor glycaemic control (n = 75), while there were no differences in Fat Mass Index (*p* = 0.0936). Cases with good glycaemic control had a higher mean value of Phase angle than those with poor glycaemic control (*p* = 0.0051).

**Figure 1:**
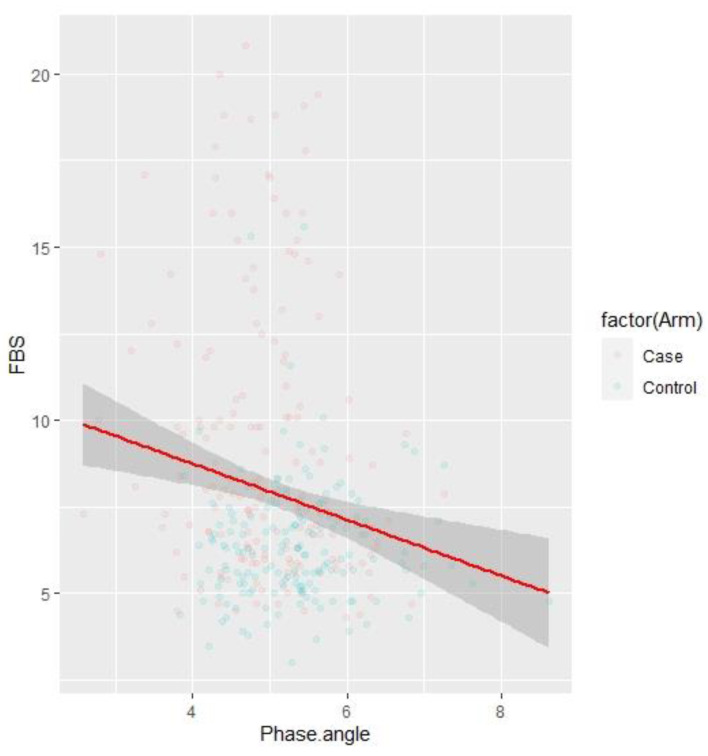
Correlation between Phase Angle and Fasting blood glucose among cases and controls.

**Figure 2:**
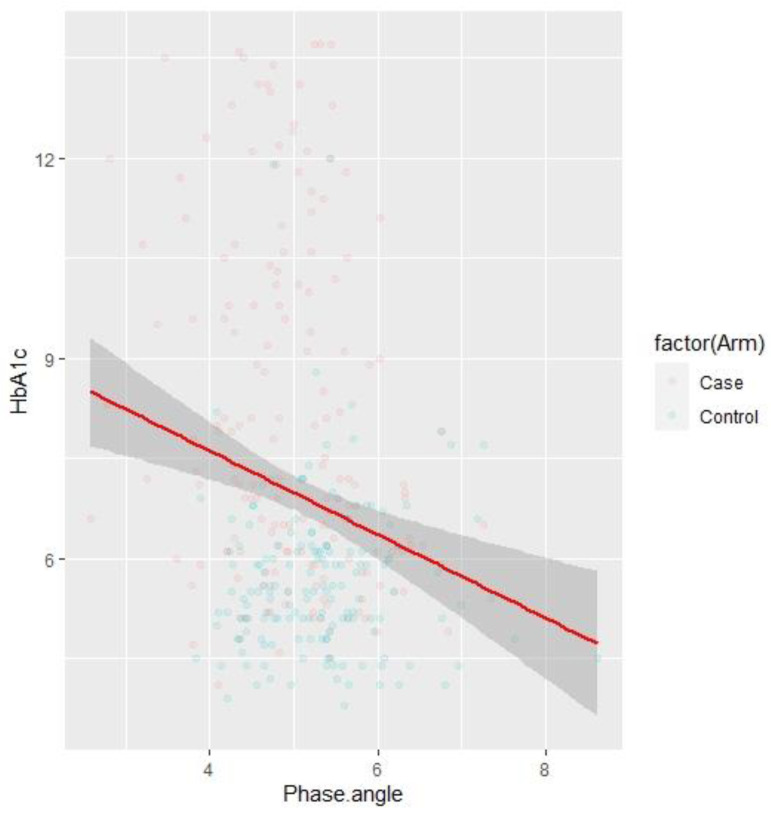
Correlation between Phase Angle and Glycated Haemoglobin.

We observed negative correlations (Figure 1) between phase angle and Fasting blood glucose (r = −0.19, *p* = 0.0004). A similar negative correlation (Figure 2) was observed between Phase Angle and glycated haemoglobin (r = −0.22, *p* < 0.001).

Body composition parameters in relation to the level of glycaemic control are summarised in Table 6.

Cases using Mixtard insulin had a higher mean Fat Mass Index than those on Insulatard and Actrapid (*p* = 0.001). We did not find evidence that the Fat Mass Index among cases differed by the duration of insulin use (*p* = 0.409), family history of diabetes (*p* = 0.244), or dietary intake scores (*p* = 0.824). Females showed higher values of Fat Mass Index than their male counterparts. Among female cases, menstruation was associated with higher values of fat mass index (mean difference = 0.88, *p* < 0.001) than females before puberty.

## 4. Discussion

The primary objective of this study was to assess whether any differences existed in the body composition characteristics of diabetic children compared to their age and sex-matched controls. In our sample, the anthropometric and bioelectrical characteristics of cases were indicative of alterations. The mean values for waist-hip ratio, skin fold circumferences, fat mass index, impedance and phase angle were significantly different from those of controls. Cases had characteristically higher central adiposity than controls. This is particularly so given that weight changes have been reported as a common side effect of intensive insulin therapy [6,19]. We have illustrated alterations in body composition among young diabetics with higher Fat Mass Index and increased central adiposity as compared to their closely matched controls. The lipogenic effect of insulin [4] on body composition is evident in our diabetic children and adolescents sample.

Body composition alterations among diabetic patients who are historically described as lean [26] is an undesirable outcome [3] of diabetes management, especially central abdominal adiposity that is associated with dyslipidaemia and insulin resistance [29]. These adverse weight changes, coupled with insufficient glycaemic control, as demonstrated by high mean HBA1c levels, place diabetic children at an increased risk of diabetic macro and microvascular complications, metabolic syndrome and double diabetes [19] if not timely addressed.

Our study findings showed that while weight and height did not differ between groups, body composition parameters did. This illustrates inaccuracies associated with the use of height, weight and their derived index of Body Mass as a body composition assessment method. BMI does not disaggregate surrogate body weight into its compartments [7]. Given the lack of valid prediction regression equations for our population, there is a scientific gap that warrants urgent research if we are to integrate BIA measurements into routine medical practice. Additionally, we could not construct Resistance: Reactance (RXc) graphs to illustrate vector positions and displacement on planes as described by [30]. The use of Bioelectrical Vector Analysis is compromised by the unavailability of suitable tolerance eclipses with which comparisons can be made [31,32]. Together, the unavailability of prediction equations is a major limitation to the wider application of BIA measurements in routine clinical practice in Uganda. However, our findings still provide valuable information regarding the body composition characteristics that can form a basis for future studies.

We have shown that diabetic participants had a higher mean value of resistance and the lower mean value of reactance, showing an increased tendency for adiposity among T1DM children. These findings are supported by scientific evidence that links abdominal obesity to insulin resistance and diabetes [33]. It is, therefore, critical to understand how T1DM, insulin hormonal therapy and adiposity are associated in order to address cardiometabolic risk factors early on, which can revert life-threatening complications.

We observed significant differences in waist-hip ratios, biceps, triceps, subscapular and suprailiac skin folds, fasting blood glucose, and glycated haemoglobin means. These results mirrored those reported previously [34,35], with diabetic children showing higher mean values of skinfolds. High HbA1c values reflect a general lack of tight glycaemic control among the diabetic children in our sample. This corresponded to the lower phase angle observed among diabetic children as similarly reported by [10,21,36]. Bioelectrical phase angle represents a clinically important indicator independent of regression algorithms and assumptions that can be used as a diagnostic tool and a marker of cell mass and general health status [37,38].

We observed a high percentage (45.7%) of diabetic cases having poor glycaemic control, yet most of the participants (83.54%) had an acceptable level of adherence and were up to date with their hospital appointments with no missed visits (76.8%). In our sample, poor glycaemic control could probably be indicative of insulin resistance due to central adiposity among these diabetic participants coupled with higher waist-hip ratios, fat mass index and skinfold circumferences. As scientific evidence suggests, good metabolic control during childhood and adolescence is highly associated with and protective against developing complications in later life, such as macroalbuminuria and retinopathy in adulthood [39]. Comparison of our findings with other similar findings is a challenge given the differences that exist in body composition machines, and measurement protocols; for instance, the use of two-electrode or four-electrode devices complicates comparisons between studies’ findings.

Taken altogether, there is evidence that our sample of diabetic children is showing early signs of insulin resistance. This underscores the need for continuous body composition monitoring among diabetes patients to support the early identification of high-risk patients with a host of cardiometabolic risk markers, put in place control measures and individually support these children. As reported by [40], among African children, there is a continuing trend of increased adiposity among diabetic children who were once thought to be lean. Our findings are particularly relevant to clinicians and medical practitioners working with T1DM children and adolescents to pay particular attention to weight changes and central adiposity during the treatment and management of diabetes. Physical activity training programs, targeted health education and routine dietetic advice are recommended.

## 5. Conclusions

The lower phase angle and high Fat Mass Index demonstrated an undesirable nutritional and body functional status of T1DM children and adolescents compared to their healthy matched controls in our sample. Given that the primary objective in managing diabetes is to reduce high serum glucose levels and prevent the onset of diabetes-related complications, monitoring body composition can aid in the early detection of changes at the cellular level. The results of this study have shown statistically significant differences in body composition among T1DM patients and their closely matched controls. We hope that our findings will form a basis for further research regarding the body composition of children in Uganda and Sub-Saharan Africa at large.

## Figures and Tables

**Table 1:** The socio-demographic characteristics of respondents.

Characteristic (s)	Cases		Controls		Total	
Socio-Demographics	Frequency n = 164	Percentage (100%)	Frequency n = 164	Percentage (100%)	Frequency n = 328	Percentage (100%)
Sex						
Male	82	50	79	48.2	161	49.1
Female	82	50	85	51.8	167	50.9
Age (years)						
6–10	31	18.9	29	17.7	60	18.3
10.1–15	58	35.4	72	43.9	130	39.6
15.1–17.9	75	45.7	63	38.4	138	42.1
Birth Order ^1^						
Firstborn	43	26.2	27	16.5	70	21.3
Second born	41	25	51	31.1	92	28
Third born	31	18.9	34	20.7	65	19.8
Forth born	25	15.2	20	12.2	45	13.7
Fifth born & above	24	14.6	31	18.9	55	16.8
Nature of residence						
Urban	132	80.5	140	85.4	272	82.90%
Rural	32	19.5	24	14.6	56	17.10%
Family size						
1–5 members	76	46.3	47	28.7	19	5.80%
6–10 members	75	45.7	108	65.9	183	55.80%
11–15 members	12	7.3	7	4.3	123	37.50%
≥15 members	1	0.6	2	1.2	3	0.90%
HIV Status						
Negative	154	93.9	153	93.3	307	93.6
Positive	8	4.9	8	4.9	16	4.9
Unknown	2	1.2	3	1.8	5	1.5
Sleep difficulties						
No	150	91.5	158	96.3	308	93.90%
Yes	14	8.5	6	3.7	20	6.10%
Wake up difficulties						
No	149	90.9	160	97.6	309	94.20%
Yes	15	9.1	4	2.4	19	5.80%

^1^ missing value.

**Table 2:** Medical history of cases.

Attribute	Number (n = 164)	Percentage (%)
Family History of Diabetes		
No	122	74.40%
Yes	42	25.10%
Family History of Hypertension		
No	130	79.30%
Yes	34	20.70%
HIV Status		
Negative	156	95.10%
Positive	8	4.90%
Insulin Infusion		
Multiple Daily Injections	164	100%
Glycaemic control		
Controlled (<7.5 HbA1c)	89	54.30%
Uncontrolled (≥7.5 HbA1c)	75	45.70%

**Table 3:** Anthropometry.

Parameter	Cases (n = 164)		Controls (n = 164)		Total Cases (n = 164)	Total Controls (n = 164)	*p* Value ^1^
	Male (n = 82)	Female (n = 82)	Male (n = 79)	Female (n = 85)			
	Mean (SD)	Mean (SD)	Mean (SD)	Mean (SD)	Mean (SD)	Mean (SD)	
Weight (Kg)	44.26 (13.14)	44.35 (15.83)	41.37 (14.40)	45.10 (11.25)	44.31 (14.50)	43.2 (12.97)	0.508
Height (cm)	154.39 (16.50)	148.60 (13.74)	154.78 (18.04)	155.25 (12.99)	151.50 (15.41)	153.6 (15.70)	0.228
BMI (Kg/m^2^)	18.10 (2.77)	19.53 (4.92)	17.31 (2.71)	18.40 (2.75)	18.81 (4.04)	17.87 (2.80)	0.0428
Sitting height (cm)	87.55 (18.30)	89.48 (15.88)	75.90 (13.1)	78.42 (8.98)	88.51 (17.09)	77.2 (11.20)	<0.001
Leg length (cm)	75.57 (25.98)	73.73 (25.10)	89.29 (12.37)	96 (10.10)	74.65 (25.50)	92.7 (11.70)	<0.001
Hip (cm)	78.06 (13.70)	78.89 (16.70)	82.67 (10.45)	87.87 (10.22)	78.50 (15.23)	85.3 (10.60)	<0.001
Waist (cm)	66.96 (12.10)	68.79 (14.22)	70.39 (7.70)	71.30 (6.45)	67.90 (13.20)	70.8 (7.08)	0.0095
Waist Hip Ratio	0.86 (0.05)	0.88 (0.06)	0.86 (0.06)	0.82 (0.06)	0.87 (0.05)	0.84 (0.06)	<0.001
MUAC (cm)	22.01 (3.22)	22.64 (4.40)	21.41 (3.34)	21.57 (2.73)	22.3 (3.85)	21.47 (3.04)	0.0265
Biceps (mm)	18.57 (5.32)	19.7 (6.10)	15.98 (4.21)	15.71 (3.84)	19.14 (5.71)	15.84 (4.01)	<0.001
Triceps (mm)	16.87 (4.96)	17.01 (5.07)	13.86 (2.70)	13.75 (3.11)	16.94 (5.00)	13.8 (2.01)	<0.001
Subscapular (mm)	17.56 (3.56)	18.38 (5.28)	15.54 (3.44)	14.89 (3.68)	17.97 (4.51)	15.20 (3.57)	<0.001
Suprailiac (mm)	18.17 (4.96)	19.51 (5.83)	13.80 (2.93)	14.61 (3.05)	18.84 (5.44)	14.22 (3.0)	<0.001

^1^ Based on Analysis of Variance with a block-matched design. MUAC: Mid Upper Arm Circumference, cm: centimetres, mm: millimetres.

**Table 4:** Summarises the metabolic profiles of cases and controls.

	Cases (n = 164)	Controls (n = 164)	*p* Value ^1^
	Mean (SD)	Mean (SD)	
Log_Fat Mass Index (Kg/m^2^)	1.08 (0.72)	0.86 (0.63)	0.0038
Fat-Free Mass Index (Kg/m^2^)	15.08 (2.28)	15.0 (2.08)	0.746
Systolic (mmHg)	108.76 (14.06)	122.56 (12.10)	<0.001
Diastolic (mmHg)	70.48 (10.36)	81.8 (8.60)	<0.001
Pulse rate (bpm)	84.85 (14.24)	76.53 (8.76)	<0.001
Log_FBS (mmol/L)	2.14 (0.4)	1.83 (0.25)	<0.001
Log_HbA1c (%)	2.04 (0.31)	1.72 (0.19)	<0.001
IDDS	6.59 (1.74)	6.50 (1.46)	0.632
Physical Activity	3.32 (1.21)	3.06 (1.17)	0.0456

^1^ Based on Analysis of Variance with a block-matched design. FBS: Fasting Blood Glucose, HbA1c: Glycated Haemoglobin, IDDS: Individual Dietary Diversity Scores.

**Table 5:** Shows the bioelectrical characteristics.

	Cases (n = 164)	Controls (n = 164)	*p* Value ^1^
	Mean (SD)	Mean (SD)	
Reactance (Ω)	52.1 (9.7)	54.6 (10.9)	0.0329
Resistance (Ω)	608.9 (86.9)	585.6 (83.2)	0.0133
Xc/Ht (Ω/m)	34.7 (7.3)	35.9 (8.3)	0.1640
R/Ht (Ω/m)	408.3 (83.2)	387.5 (80.2)	0.0215
Impedance (Ω)	611.1 (87.1)	588.2 (82.9)	0.0145
PhA(Degrees)	4.94 (0.81)	5.32 (0.80)	<0.001

^1^ Based on Analysis of Variance with a block-matched design. Xc: Reactance, R: Resistance, Ht: Height(m), Xc/Ht: Reactance adjusted by Height (m). R/Ht: Resistance adjusted by Height (m) PhA: Phase angle.

**Table 6:** Shows the differences in body composition by glycaemic control.

	Uncontrolled (≥7.5 HbA1c)	Controlled (<7.5 HbA1c)	*p* Value ^1^
	Mean	Mean	
Log_FMI	1.180	0.989	0.0936
Fat-Free Mass Index	14.614	15.473	0.0143
Impedance (Ω)	626.97	597.79	0.0308
PhA(Degrees)	4.7545	5.0997	0.0051

^1^ Based on an independent samples *t*-test with unequal variances.

## Data Availability

The datasets generated during and/or analysed during the current study are available from the corresponding author on reasonable request.
